# Clinical, biological and cytometric characteristics of two patients with a homozygous A91V *PRF1* mutation

**DOI:** 10.1002/cti2.70081

**Published:** 2026-03-15

**Authors:** Nicolas Perrard, Claire Poggi, Sébastien Sanges, Bruno Lemarchant, Wadih Abou Chahlah, Louis Terriou, Stéphanie Delangue, Jacques Trauet, Myriam Labalette, Eric Hachulla, Despina Moshous, Guillaume Lefevre, David Launay

**Affiliations:** ^1^ Department of Internal Medicine and Clinical Immunology CHU Lille Lille France; ^2^ Institute for Translational Research in Inflammation Univ. Lille, Inserm, CHU Lille, U1286 ‐ INFINITE Lille France; ^3^ Regional Center for Primary Immune Deficiency CEREDIH, CHU Lille Lille France; ^4^ Department of Neurology CRC‐SEP, CHU Lille Lille France; ^5^ LilNCog ‐ Inserm U1172 Lille France; ^6^ Department of Paediatric Haematology CHU Lille Lille France; ^7^ Paediatric Intensive Care Unit CHU Lille Lille France; ^8^ Institute of Immunology CHU Lille Lille France; ^9^ Pediatric Immunology, Hematology and Rheumatology Necker‐Enfants Malades University Hospital, AP‐HP Paris France; ^10^ Laboratory of Genome Dynamics in the Immune System Imagine Institute, INSERM UMR Paris France; ^11^ Necker‐Enfants Malades University Hospital, AP‐HP, CEREDIH Paris France

**Keywords:** familial haemophagocytic lymphohistiocytosis, haemophagocytic lymphohistiocytosis, perforin/granzyme system, primary immunodeficiency

## Abstract

**Objectives:**

Inborn errors of immunity are rare genetic disorders that cause dysfunction of the immune system. Among these, familial haemophagocytic lymphohistiocytosis (FHL) involves defects in the perforin/granzyme pathway, which is essential for regulating immune responses. These conditions predispose individuals to haemophagocytic lymphohistiocytosis, a life‐threatening hyperinflammatory syndrome. FHL has traditionally been described in paediatric patients with a fatal outcome in the absence of early haematopoietic stem cell transplantation. However, some patients harbouring missense mutations may present with atypical or late‐onset symptoms, for which management remains unstandardised. Among these reported variants, the pathogenicity of the A91V *PRF1* mutation remains the most controversial in the literature.

**Method:**

We report clinical, biological and cytometric characteristics of two patients with a homozygous *PRF1* A91V mutation who developed clinical features consistent with FHL2.

**Discussion:**

We discuss the latest 2024 classifications to better characterise this group of disorders and their underlying genetic basis, with particular emphasis on atypical presentations and the A91V mutation, which may pose diagnostic and therapeutic challenges.

**Conclusion:**

A91V *PRF1* variant appears to represent a risk factor for the development of atypical FHL manifestations under divers triggers.

## Graphical abstract

Familial haemophagocytic lymphohistiocytosis (FHL) is a rare, inherited granzyme–perforin system defect that predisposes individuals to haemophagocytic lymphohistiocytosis. This article presents two original case reports of FHL type 2, along with a comprehensive literature review. Together, these elements aim to enhance the current understanding of FHL and guide the management of these severe and life‐threatening conditions.

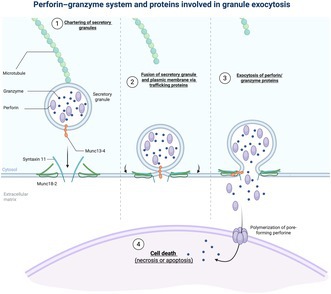

## Introduction

Inborn errors of immunity encompass a group of rare pathologies that predispose affected individuals to a variety of infections, autoimmune diseases and malignancies.^1^

The perforin–granzyme system is a cytotoxic protein system contained within the lysosomes of cytotoxic T lymphocytes (CD3^+^ CD8^+^) or NK cells (CD16^+^ and/or CD56^+^), which facilitates the rapid destruction of target cells within minutes and immune system homeostasis. Defects in perforin transcription were first described in 1999 by Stepp *et al*.^2^ and are responsible for the early onset of haemophagocytic lymphohistiocytosis (HLH) during childhood. This group of diseases was then named familial haemophagocytic lymphohistiocytosis (FHL).^3^

This article aims to report clinical, biological and cytometric characteristics of two patients diagnosed in our centre with atypical FHL2 and discuss the current classifications of FHL with a particular focus on partial defects in the perforin–granzyme system and A91V missense mutation in the *PRF1* gene which encodes perforin.

## Results

### Clinical and biological descriptions

The first case, a 1‐year‐old child, presented with a primary varicella‐zoster virus (VZV) infection accompanied by neurological involvement. The clinical examination revealed meningeal stiffness, and neurological assessment showed abnormalities in brainstem reflexes, dysautonomia and a bilateral pyramidal syndrome, all suggestive of meningoencephalitis, which required immediate transfer to the intensive care unit. Shortly after admission, the patient developed HLH, with fever, hepatosplenomegaly, bicytopaenia (anaemia 6.8 g dL^−1^ and thrombocytopenia 74 × 10^9^ L^−1^), hypofibrinogenaemia at 1.5 g L^−1^ and hypertriglyceridemia at 4.74 g L^−1^. Ferritin and LDH levels were mildly elevated at 404 ng mL^−1^ (*N*: 20–300 ng mL^−1^) and 378 U L^−1^ (*N*: 120–246 U L^−1^), respectively. Bone marrow analysis did not show signs of haemophagocytosis.

Neurological investigations included cerebrospinal fluid (CSF) analysis showing four leukocytes per mm^3^, with normal protein and glucose levels. PCR for VZV was negative in CSF, as well as PCR for HSV 1 & 2 and enteroviruses. Brain MRI was normal, while the initial electroencephalogram was suggestive of meningoencephalitis. The overall findings were not highly suggestive of VZV meningoencephalitis, leading to the consideration of HLH‐related neurological involvement. Therapeutic management included antiviral therapy with acyclovir 250 mg every 8 h for 14 days, corticosteroid therapy with methylprednisolone pulses (1 mg kg^−1^) for 2 days, followed by oral prednisone (1 mg kg^−1^ day^−1^), which led to a rapid clinical improvement and did not require any additional HLH‐specific therapies. Given the patient's young age, a possible atypical FHL was then suggested.

The second case was a 44‐year‐old adult patient with a history of testicular seminoma at age 29 and asymptomatic splenic nodular lesions and bilateral mediastinal lymphadenopathy with uptake on PET‐CT (SUV between 4 and 6) found during neoplasia remission monitoring. At age 44, the patient was admitted to our centre due to the sudden onset of fever and spastic tetraparesis, pancytopenia (haemoglobin 8 g dL^−1^, platelets 46 × 10^9^ L^−1^ and neutrophils 0.8 × 10^9^ L^−1^), hypofibrinogenaemia at 1.8 g L^−1^ and moderate hypertriglyceridemia at 2.49 g L^−1^. Ferritin levels were elevated at 6854 ng mL^−1^ (*N*: 20–300 ng mL^−1^) and LDH at 701 U L^−1^ (*N*: 120–246 U L^−1^), with no signs of haemophagocytosis found in bone marrow analyses. Brain MRI revealed a non‐specific FLAIR hyperintensity in the right cerebellar peduncle. PET‐CT revealed an increase in the intensity and extent of splenic hypermetabolism, which were diffuse (SUV at 17), with spleen size increasing to 21 cm. Extensive evaluation for secondary HLH was negative. During inpatient care, the patient developed worsening HLH‐related biological markers, episodes of hemodynamic instability, and suspecting splenic lymphoma, splenectomy was promptly performed but histopathologic analysis only showed signs of haemophagocytosis and lymphohistiocytic infiltrates resembling loose granulomas with no evidence of neoplasia. The patient's condition improved immediately after the splenectomy without any additional therapies. We observed a complete regression of the spastic tetraparesis and HLH biological markers. Follow‐up remained uneventful for 4 years.

**Figure 1:**
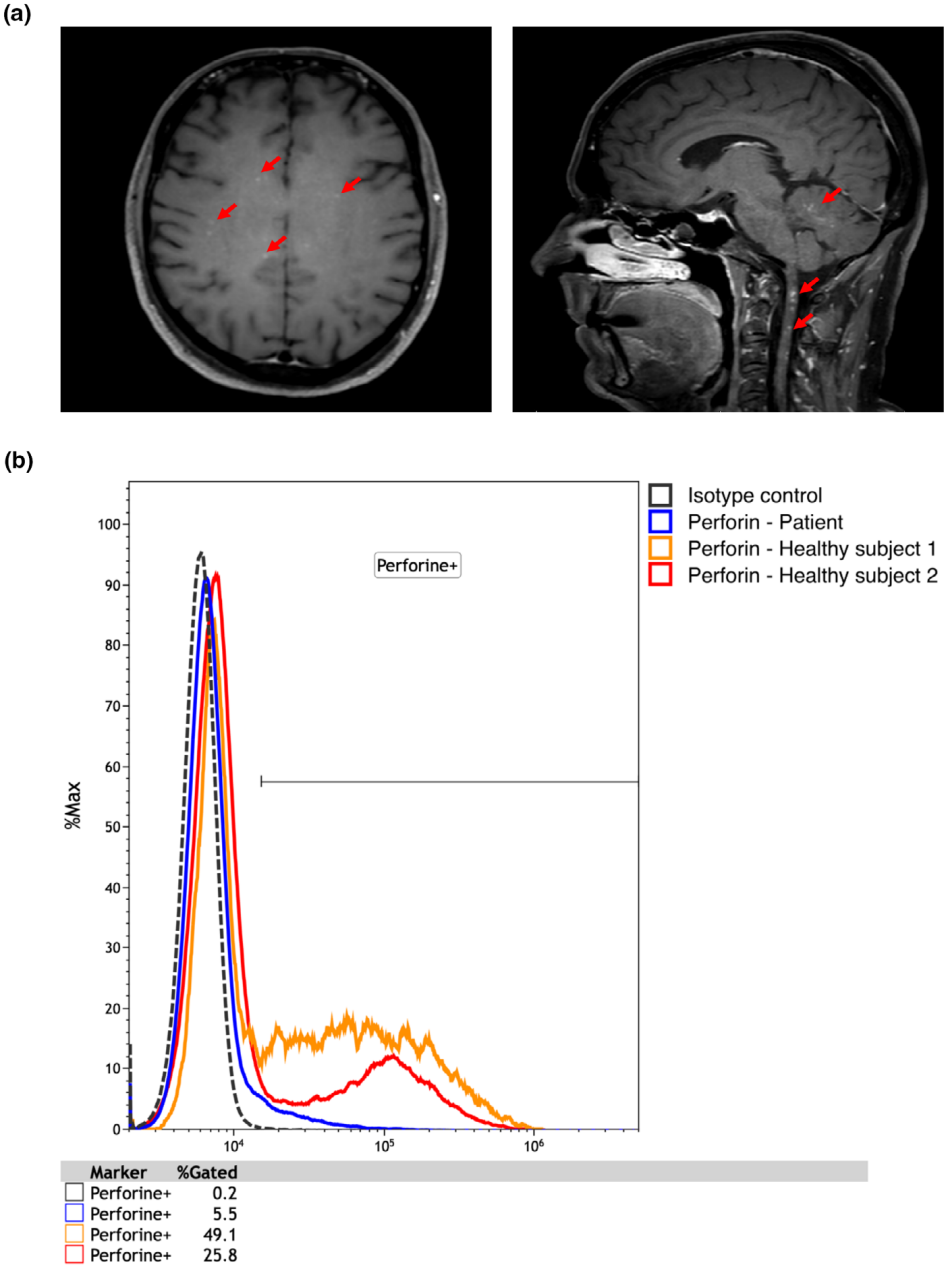
Sagittal and axial T1‐weighted MRI images with gadolinium contrast showing numerous punctate parenchymal enhancements scattered across supratentorial, infratentorial and medullary regions, particularly in the right middle cerebellar peduncle (red arrows) **(a)**. Monoparametric histogram showing a relative decrease in perforin expression by flow cytometry in CD8^+^ T lymphocytes for the adult patient (blue plot) compared to two healthy subjects (red and orange plots). Dotted‐black plot represents flow cytometry isotype control **(b)**.

At age 48, the patient was admitted to the Neurology Department because of rapid apparition of spastic tetraparesis. Brain and spinal MRI revealed diffuse punctate parenchymal contrast enhancements, suggestive of CLIPPERS syndrome (chronic lymphocytic inflammation with pontine perivascular enhancement responsive to steroids) or cerebro‐meningeal granulomatosis (Figure 1a).^4^ No HLH biological blood markers were present, and CSF analysis showed one leukocyte per mm^3^, with no hyperproteinorachia or hypoglycorrhachia, and two oligoclonal bands on CSF isoelectric focusing. Given the clinical presentation at the age of 44 years, a transbronchial biopsy of mediastinal lymphadenopathy was performed and revealed lymphohistiocytic infiltrates resembling loose granulomas, supporting the diagnosis of sarcoidosis with neurological and lymph node involvement. Initial treatment included subcutaneous methotrexate (0.3 mg kg^−1^ week^−1^) and a slowly tapering dose of corticosteroids (0.5 mg kg^−1^). However, due to a recurrence of neurological symptoms, methotrexate was replaced by intravenous infliximab, leading to complete resolution of neurological symptoms and stability of metabolic lesions on PET‐CT.

At age 52, the patient experienced a neurological relapse with the re‐emergence of gait disturbances and signs of intracranial hypertension. Brain and spinal MRI confirmed the reappearance of multiple contrast‐enhancing lesions in the subcortical and deep supratentorial and infratentorial white matter. No HLH biological blood markers were present. Given the personal history of HLH, the atypical evolution of CLIPPERS syndrome with steroid dependence, and the progression of neurological symptoms under anti‐TNFα therapy, a diagnosis of atypical FHL was subsequently considered.

### Cytometric and genetic characteristics

For both patients, cytometric immunological investigations were performed, identifying B lymphopenia, with activation of the circulating T CD8^+^ lymphocyte population based on HLA‐DR membrane expression. Flow cytometric analyses were performed in the first case during the HLH episode and in the second case at age 52 during the neurological deterioration associated with CLIPPERS syndrome. Perforin/granzyme system flow cytometry analyses showed present but reduced perforin expression in T CD8^+^ and NK lymphocytes from both patients compared to positive controls (Figure 1b).

Genetic analyses confirmed the presence of a c.272C>T (p.(Ala91Val)) mutation in exon 2 of the *PRF1* gene in a homozygous state, verified by Sanger sequencing for both the patients and the parents of the youngest patient, who were heterozygous carriers.

### Follow‐up

During follow‐up, there was no recurrence of HLH despite two documented rhinovirus infections and asymptomatic EBV seroconversion in the first patient.

A second flow cytometric evaluation, performed 2 years after diagnosis outside of any HLH episode, confirmed reduced perforin expression in CD8^+^ T lymphocytes and NK cells compared with positive controls, with disappearance of the circulating CD8^+^ T‐cell population expressing surface HLA‐DR^+^.

For the adult patient, treatment with rituximab was initiated based on literature evidence. This therapeutic change led to a favorable outcome, with complete regression of neurological symptoms and resolution of MRI lesions at the 1‐year follow‐up.

## Discussion

We present two cases of atypical FHL2 in paediatric and adult care settings. Both patients initially presented with HLH, the classical presentation of FHL2. Nevertheless, the evolution was atypical in both cases. In the paediatric case, HLH resolved following corticosteroid therapy and symptomatic intensive care measures, while in the adult case, splenectomy led to HLH resolution without specific treatment. Interestingly, allogeneic haematopoietic stem cell transplantation (HSCT) was not performed in either patient, despite HSCT being considered the only curative treatment for HLH in the context of FHL, with other treatments being merely temporary measures used as a bridge to HSCT. The adult patient subsequently developed neurological manifestations consistent with CLIPPERS syndrome or neurosarcoidosis. However, the atypical disease course characterised by steroid dependence, as well as anti‐TNFα therapy, and the rare association with HLH were inconsistent with sarcoidosis and ultimately supported a diagnosis of atypical FHL.

Both patients exhibited detectable perforin expression in NK cells and CD8^+^ T lymphocytes, albeit at reduced intensity compared with a positive control. Analysis of the activated CD8^+^ HLA‐DR^+^ T‐cell population revealed disappearance of this subset at a distance from symptom onset, consistent with the clinical and biological resolution of symptoms.

**Figure 2:**
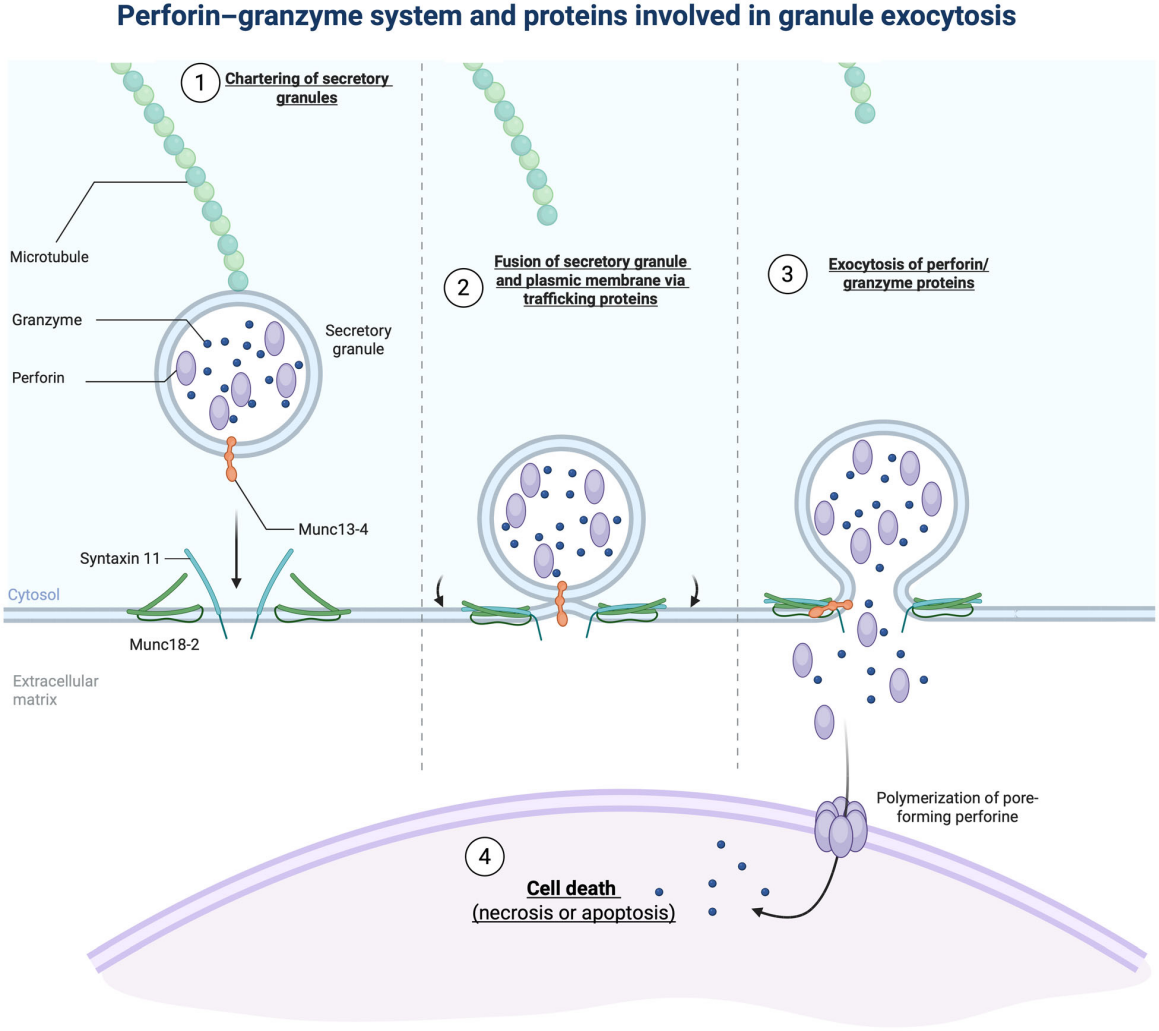
Diagram of mechanisms and molecules involved in cytotoxic granule exocytosis, highlighting their roles in the pathogenesis of familial haemophagocytic lymphohistiocytosis.

Mutations in the gene encoding perforin are responsible for FHL, but there are also various proteins involved in the transport of cytotoxic lysosomes to the plasma membrane, their fusion and the exocytosis of granule contents. These proteins have also been described in diseases associated with defects in this system,^5^ such as Munc13‐4 (*UNC13D* gene), Syntaxin 11 (*STX11* gene) and Munc18‐2 (*STXBP2* gene), respectively, involved in FHL3, FHL4 and FHL5 (Table 1, Figure 2).^6^, ^7^, ^8^, ^9^.

**Table 1:** Summary of the different causes of familial haemophagocytic lymphohistiocytosis

Name	Percentage of total FHL (%)	OMIM number	Mutated gene	Locus	Coded protein
FHL 2	30–40	267700	*PRF1*	9q21.3–q22	Perforin
FHL 3	20–40	608898	*UNC13D*	17q25.1	Munc13‐4
FHL 4	5–25	603552	*STX11*	6q24	Syntaxin11
FHL 5	5–25	613101	*STXBP2*	19p13	Munc18‐2

FHL, familial haemophagocytic lymphohistiocytosis.

The classic presentation of FHL corresponds to the early onset, during the first months of life, of HLH as defined in the HLH‐2004 studies by the Histiocyte Society. An infectious trigger, often mild, is sometimes identified in these children but can be absent.^10^ In 2024, a re‐evaluation of the HLH‐2004 diagnostic criteria confirmed their excellent diagnostic validity as currently applied and proposed two complementary alternative diagnostic pathways: a genetic pathway, involving the identification of pathogenic genetic variants, and a cellular pathway, including absence of perforin expression, impaired NK‐cell degranulation and cytotoxicity assays (Table 2). The authors nevertheless emphasise the absolute necessity of a comprehensive etiological approach to HLH and the exclusion of secondary causes. These criteria have not been tested for discriminating FHL from secondary HLH and therefore cannot be extrapolated to populations other than those studied.^11^

**Table 2:** Revised diagnostic guidelines for familial haemophagocytic lymphohistiocytosis, from Henter *et al.* (2004^10^ and 2024^11^)

*The diagnosis HLH can be established if at least 1 of either (1), (2) or (3) below is fulfilled*
(1) A molecular diagnosis consistent with HLH in a patient with signs/symptoms suggestive of HLH
(2) Functional cellular findings consistent with FHL in a patient with signs/symptoms suggestive of HLH
(3) Diagnostic criteria for HLH fulfilled (at least five out of the eight criteria below) AND no evidence of malignancy Fever ≥ 38.5°C Splenomegaly (≥ 2 cm below the costal margin) Cytopaenias (affecting ≥ 2 of 3 lineages in the peripheral blood): Haemoglobin < 90 g L^−1^ (in infants < 4 weeks: haemoglobin < 100 g L^−1^) Platelets < 100 × 10^9^ L^−1^ Neutrophils < 1.0 × 10^9^ L^−1^ Hypertriglyceridemia and/or hypofibrinogenaemia: Fasting triglycerides ≥ 3.0 mmol/L (i.e. ≥ 265 mg dL^−1^) Fibrinogen ≤ 1.5 g L^−1^ Haemophagocytosis in bone marrow or spleen or lymph nodes Ferritin ≥ 500 μg L^−1^ Soluble CD25 (i.e. soluble IL‐2 receptor) ≥ 2400 U mL^−1^

Comments: If haemophagocytic activity is not proven at the time of presentation, further search for haemophagocytic activity is encouraged. If the bone marrow specimen is not conclusive, material may be obtained from other organs. Serial marrow aspirates over time may also be helpful. The following findings may provide strong supportive evidence for the diagnosis: (a) spinal fluid pleocytosis (mononuclear cells) and/ or elevated spinal fluid protein, (b) histological picture in the liver resembling chronic persistent hepatitis (biopsy). Other abnormal clinical and laboratory findings consistent with the diagnosis are: cerebro‐meningeal symptoms, lymph node enlargement, jaundice, oedema, skin rash. Hepatic enzyme abnormalities, hypoproteinaemia, hyponatremia, high VLDL, low HDL. It is always of vital importance to search for the underlying cause(s) for the HLH syndrome, which may be either genetic, such as FHL, and/or acquired, such as an infection, a malignancy or an autoimmune or autoinflammatory disorder.

Following the seminal description in 1999, the first cases of adult patients harbouring homozygous mutations in the perforin gene were reported. These late‐onset cases are often atypical, not necessarily presenting with classic HLH symptoms, but rather with neurological and pulmonary involvement, lymphoma or autoimmune diseases, as described in a small series of 10 patients collected in 2015.^12^ These patients carried biallelic *PRF1* mutations including at least one missense variant, with perforin expression often detectable in NK and T cells. Notably, a patient who presented with pulmonary involvement followed by HLH underwent splenectomy, and no recurrence of HLH was observed thereafter, like the case we report here and several other HLH cases described in the literature.^13^

Neurological involvement appears to be a common feature in these late‐onset forms of FHL2. In 2020, Blincoe *et al*. described 38 paediatric and adult patients with central nervous system involvement and a diagnosis of primary HLH, including 23 FHL2 patients. Brain biopsies revealed lymphohistiocytic infiltration and loose granulomas, suggestive of ‘localized neurological HLH’, according to the authors. Among the 38 patients, 19 had undergone HSCT, resulting in stability or improvement of neurological involvement in 80% of cases, while 67% of the 15 non‐transplanted patients died. Three patients were treated with rituximab, leading to subsequent improvement in their neurological condition without HSCT, like the adult case we report.^14^ In 2021, Taieb *et al*. reported the investigation of 12 patients diagnosed with a CLIPPERS syndrome resulting in the diagnosis of three FHL2 and one FHL3 patients. All three FHL2 patients harboured biallelic *PRF1* mutations, which were consistently missense variants, with the presence of perforin in NK cells during biological testing. The CLIPPERS syndrome was atypical in 75% of cases, particularly regarding steroid dependence, and the radiological evolution with the appearance of confluent contrast‐enhancing lesions during the second CLIPPERS flare‐up.^4^, ^15^

A91V, the single amino acid change resulting from the c.272C>T substitution in exon 2, is by far the most widely debated *PRF1* missense mutation concerning its pathogenicity in the homozygotic state. According to the GnomAD database, this mutation is considered a neutral polymorphism or potential risk factor found in 6.8% of tested individuals in the heterozygotic state, with more than 1000 individuals in the homozygous state. Cases of healthy homozygous individuals for *PRF1* A91V have also been reported, supporting this mutation as a neutral polymorphism.^16^ Analysis of large databases (GnomAD and the population‐based UK Biobank) and the prospective ASPREE cohort did not appear to show an increased immune‐mediated diseases or mortality in individuals with homozygous *PRF1* A91V or in heterozygotes carrying this variant alongside another missense or truncating mutation, subject to the recruitment and selection biases inherent to such cohorts.^17^, ^18^

However, this polymorphism of *PRF1* has been described as a factor associated with the occurrence of various diseases at the heterozygous state, including BCR‐ABL acute lymphoblastic leukaemia and T/NK intranasal lymphomas,^19^ multiple sclerosis and type 1 diabetes,^20^, ^21^ or severe forms of SARS‐CoV‐2 infections.^22^ Several independent studies have established that this single amino acid change severely affects the level and function of the perforin protein *in vitro*, emphasising that the alanine at position 91 is conserved in the genes encoding human, mouse and rat perforins.^23^, ^24^ Several case reports have described atypical FHL2 in patients homozygous for *PRF1* A91V, as well as in compound heterozygotes carrying this variant in combination with another missense or truncating mutation.^15^, ^25^, ^26^, ^27^, ^28^ In 2020, Jang *et al*.^28^ reported a homozygous *PRF1* A91V patient who developed EBV‐triggered HLH, with impaired NK‐cell cytotoxicity that normalised 1 and 2 months after clinical resolution. Regarding the two cases we reported, functional cytotoxicity assays could not be performed at the time of the HLH episodes, which constitutes a limitation in interpreting the direct involvement of the PRF1 A91V variant in HLH onset.

Collectively, these data, together with our observations, support the notion that the *PRF1* A91V variant may act as a genetic susceptibility factor for atypical FHL2, potentially unmasked by infectious or immune‐mediated triggers.

## Conclusion

We describe two clinical cases of patients carrying homozygous A91V *PRF1* mutation, associated with a mild or late‐onset FHL phenotype. Given the frequency of this mutation in some populations and the presence of healthy homozygous carriers, this genotype appears to represent a risk factor for the development of atypical FHL manifestations (such as mild HLH or neurological disease) under certain conditions, such as infectious, inflammatory or still unknown triggers.

Understanding this broad phenotypic spectrum is essential for clinicians as FHL diagnostic should be considered and established in adult patients presenting with compatible atypical symptoms.

## Author contributions

**Nicolas Perrard:** Conceptualization; investigation; visualization; writing – original draft; writing – review and editing. **Claire Poggi:** Conceptualization; investigation; writing – original draft. **Sébastien Sanges:** Conceptualization; investigation; supervision; validation. **Bruno Lemarchant:** Investigation; supervision; validation. **Wadih Abou Chahlah:** Resources; investigation; supervision; validation. **Louis Terriou:** Supervision. **Stéphanie Delangue:** Supervision. **Jacques Trauet:** Software; formal analysis; visualization. **Myriam Labalette:** Resources; supervision. **Eric Hachulla:** Resources; supervision. **Despina Moshous:** Investigation; resources; supervision. **Guillaume Lefevre:** Resources; investigation; supervision; validation; writing – original draft. **David Launay:** Resources; investigation; supervision; validation; writing – original draft.

## Conflict of interest

SS reports meeting fees from Shire, Sanofi‐Genzyme, SOBI, Novartis, BioCryst; consulting fees from Novartis, Takeda and Grifols; speaker fees from MSD; research grants from BioCryst, MSD, outside the submitted work. BL received personal compensations for consulting from ALEXION and congresses registration and travelling expenses from NOVARTIS, JANSSEN and UCB Pharma. GL has received honoraria for serving on the board and for participating in symposia from Takeda, LFB, Grifols and research support from LFB, Takeda, CSL Behring, Biotest, Octapharma. The rest of the authors declare they have no relevant conflicts of interest.

## Data Availability

The data that support the findings of this study are available on request from the corresponding author. The data are not publicly available due to privacy or ethical restrictions.

## References

[1] Seidel MG , Kindle G , Gathmann B *et al*. The European Society for Immunodeficiencies (ESID) registry working definitions for the clinical diagnosis of inborn errors of immunity. J Allergy Clin Immunol Pract 2019; 7: 1763–1770. 30776527 10.1016/j.jaip.2019.02.004

[2] Stepp SE , Dufourcq‐Lagelouse R , Le Deist F *et al*. Perforin gene defects in familial hemophagocytic lymphohistiocytosis. Science 1999; 286: 1957–1959. 25980028

[3] Poli MC , Aksentijevich I , Bousfiha AA *et al*. Human inborn errors of immunity: 2024 update on the classification from the International Union of Immunological Societies Expert Committee. J Hum Immun 2025; 1: e20250003. 41608114 10.70962/jhi.20250003 PMC12829761

[4] Pittock SJ , Debruyne J , Krecke KN *et al*. Chronic lymphocytic inflammation with pontine perivascular enhancement responsive to steroids (CLIPPERS). Brain 2010; 133: 2626–2634. 20639547 10.1093/brain/awq164

[5] Sieni E , Cetica V , Mastrodicasa E *et al*. Familial hemophagocytic lymphohistiocytosis: a model for understanding the human machinery of cellular cytotoxicity. Cell Mol Life Sci 2012; 69: 29–40. 21990010 10.1007/s00018-011-0835-y PMC11114696

[6] Feldmann J , Callebaut I , Raposo G *et al*. Munc13‐4 is essential for cytolytic granules fusion and is mutated in a form of familial hemophagocytic lymphohistiocytosis (FHL3). Cell 2003; 115: 461–473. 14622600 10.1016/s0092-8674(03)00855-9

[7] zur Stadt U , Schmidt S , Kasper B *et al*. Linkage of familial hemophagocytic lymphohistiocytosis (FHL) type‐4 to chromosome 6q24 and identification of mutations in syntaxin 11. Hum Mol Genet 2005; 14: 827–834. 15703195 10.1093/hmg/ddi076

[8] zur Stadt U , Rohr J , Seifert W *et al*. Familial hemophagocytic lymphohistiocytosis type 5 (FHL‐5) is caused by mutations in Munc18‐2 and impaired binding to syntaxin 11. Am J Hum Genet 2009; 85: 482–492. 19804848 10.1016/j.ajhg.2009.09.005 PMC2756548

[9] Côte M , Ménager MM , Burgess A *et al*. Munc18‐2 deficiency causes familial hemophagocytic lymphohistiocytosis type 5 and impairs cytotoxic granule exocytosis in patient NK cells. J Clin Invest 2009; 119: 3765–3773. 19884660 10.1172/JCI40732 PMC2786810

[10] Henter JI , Horne A , Aricó M *et al*. HLH‐2004: Diagnostic and therapeutic guidelines for hemophagocytic lymphohistiocytosis. Pediatr Blood Cancer 2007; 48: 124–131. 16937360 10.1002/pbc.21039

[11] Henter JI , Sieni E , Eriksson J *et al*. Diagnostic guidelines for familial hemophagocytic lymphohistiocytosis revisited. Blood 2024; 144: 2308–2318. 39046779 10.1182/blood.2024025077 PMC11619794

[12] Tesi B , Chiang SC , El‐Ghoneimy D *et al*. Spectrum of atypical clinical presentations in patients with biallelic PRF1 missense mutations: atypical presentations of perforin deficiency. Pediatr Blood Cancer 2015; 62: 2094–2100. 26184781 10.1002/pbc.25646

[13] Fouquet G , Larroche C , Carpentier B *et al*. Splenectomy for haemophagocytic lymphohistiocytosis of unknown origin: risks and benefits in 21 patients. Br J Haematol 2021; 194: 638–642. 33961306 10.1111/bjh.17497

[14] Blincoe A , Heeg M , Campbell PK *et al*. Neuroinflammatory disease as an isolated manifestation of hemophagocytic lymphohistiocytosis. J Clin Immunol 2020; 40: 901–916. 32638196 10.1007/s10875-020-00814-6

[15] Taieb G , Kaphan E , Duflos C *et al*. Hemophagocytic lymphohistiocytosis gene mutations in adult patients presenting with CLIPPERS‐like syndrome. Neurol Neuroimmunol Neuroinflamm 2021; 8: e970. 33658321 10.1212/NXI.0000000000000970 PMC7963436

[16] Solomou EE , Gibellini F , Stewart B *et al*. Perforin gene mutations in patients with acquired aplastic anemia. Blood 2007; 109: 5234–5237. 17311987 10.1182/blood-2006-12-063495 PMC1890825

[17] Voskoboinik I , Lacaze P , Jang HS *et al*. Prevalence and disease predisposition of p.A91V perforin in an aged population of European ancestry. Blood 2020; 135: 582–584. 31932842 10.1182/blood.2019003487 PMC7033372

[18] Wegehaupt O , Borisov O , Sieni E *et al*. Beyond genotype: challenges in predicting disease risk for carriers of biallelic perforin variants. Blood 2025; 145: 2992–3006. 40090000 10.1182/blood.2024027954 PMC12824689

[19] Manso R , Rodríguez‐Pinilla SM , Lombardia L *et al*. An A91V SNP in the perforin gene is frequently found in NK/T‐cell lymphomas. PLoS One 2014; 9: e91521. 24632576 10.1371/journal.pone.0091521 PMC3954696

[20] Sidore C , Orrù V , Cocco E *et al*. PRF1 mutation alters immune system activation, inflammation, and risk of autoimmunity. Mult Scler 2021; 27: 1332–1340. 33566725 10.1177/1352458520963937 PMC8044257

[21] Orilieri E , Cappellano G , Clementi R *et al*. Variations of the perforin gene in patients with type 1 diabetes. Diabetes 2008; 57: 1078–1083. 18198357 10.2337/db07-0947

[22] Cabrera‐Marante O , de Rodríguez Frías E , Pleguezuelo DE *et al*. Perforin gene variant A91V in young patients with severe COVID‐19. Haematologica 2020; 105: 2844–2846. 33256384 10.3324/haematol.2020.260307 PMC7716361

[23] Trizzino A , zur Stadt U , Ueda I *et al*. Genotype phenotype study of familial haemophagocytic lymphohistiocytosis due to perforin mutations. J Med Genet 2007; 45: 15–21. 17873118 10.1136/jmg.2007.052670

[24] Voskoboinik I , Sutton VR , Ciccone A *et al*. Perforin activity and immune homeostasis: The common A91V polymorphism in perforin results in both presynaptic and postsynaptic defects in function. Blood 2007; 110: 1184–1190. 17475905 10.1182/blood-2007-02-072850

[25] Voskoboinik I , Smyth MJ , Trapani JA . Perforin‐mediated target‐cell death and immune homeostasis. Nat Rev Immunol 2006; 6: 940–952. 17124515 10.1038/nri1983

[26] Stadermann A , Haar M , Riecke A *et al*. Late onset of primary hemophagocytic lymphohistiocytosis (HLH) with a novel constellation of compound heterozygosity involving two missense variants in the PRF1 gene. Int J Mol Sci 2024; 25: 2762. 38474010 10.3390/ijms25052762 PMC10931657

[27] Palterer B , Brugnolo F , Sieni E , Barilaro A , Parronchi P . Neuromyelitis optica, atypical hemophagocytic lymphohistiocytosis and heterozygous perforin A91V mutation. J Neuroimmunol 2017; 311: 10–13. 28863861 10.1016/j.jneuroim.2017.08.003

[28] Jang HS , Flinsenberg TWH , Lacaze P *et al*. Recovery of natural killer cell cytotoxicity in a A91V perforinhomozygous patient following severe haemophagocytic lymphohistiocytosis. Br J Haematol 2020; 190: 458–461. 32342501 10.1111/bjh.16660

